# Hot Spot Analysis of YAP-TEAD Protein-Protein Interaction Using the Fragment Molecular Orbital Method and Its Application for Inhibitor Discovery

**DOI:** 10.3390/cancers13164246

**Published:** 2021-08-23

**Authors:** Jongwan Kim, Hocheol Lim, Sungho Moon, Seon Yeon Cho, Minhye Kim, Jae Hyung Park, Hyun Woo Park, Kyoung Tai No

**Affiliations:** 1 Department of Biotechnology, Yonsei University, Seoul 03722, Korea; myulchi@yonsei.ac.kr; 2 Bioinformatics and Molecular Design Research Center (BMDRC), Incheon 21983, Korea; 3 The Interdisciplinary Graduate Program in Integrative Biotechnology and Translational Medicine, Yonsei University, Incheon 21983, Korea; hclim@bmdrc.org; 4 Baobab AiBIO Co., Ltd., Incheon 21983, Korea; shmoon@baobabaibio.com (S.M.); sycho@baobabaibio.com (S.Y.C.); mhkim@baobabaibio.com (M.K.); 5 Department of Biochemistry, College of Life Science and Biotechnology, Yonsei University, Seoul 03722, Korea; jhpark.main@gmail.com (J.H.P.); hwp003@yonsei.ac.kr (H.W.P.); 6 Institute of Convergence Science and Technology, Yonsei University, Incheon 21983, Korea

**Keywords:** protein-protein interaction, YAP-TEAD inhibitor, virtual screening, binding assays, fragment molecular orbital method, anticancer

## Abstract

**Simple Summary:**

YAP-TEAD protein-protein interaction is a crucial feature in the Hippo pathway for cancer therapeutics. In this study, to investigate novel YAP-TEAD protein-protein interaction inhibitors, the fragment molecular orbital method for hot spot analysis of YAP-TEAD PPI and FMO-based pharmacophore construction were introduced. We performed pharmacophore based virtual screening through an in-house library. Virtual hit compounds were derived, and the biological activity of the hit compounds was confirmed, through in vitro experiments. Finally, we proposed a compound BY03 as a novel promising PPI inhibitor for the YAP-TEAD interaction.

**Abstract:**

The Hippo pathway is an important signaling pathway modulating growth control and cancer cell proliferation. Dysregulation of the Hippo pathway is a common feature of several types of cancer cells. The modulation of the interaction between yes-associated protein (YAP) and transcriptional enhancer associated domain (TEAD) in the Hippo pathway is considered an attractive target for cancer therapeutic development, although the inhibition of PPI is a challenging task. In order to investigate the hot spots of the YAP and TEAD1 interacting complex, an ab initio Fragment Molecular Orbital (FMO) method was introduced. With the hot spots, pharmacophores for the inhibitor design were constructed, then virtual screening was performed to an in-house library. Next, we performed molecular docking simulations and FMO calculations for screening results to study the binding modes and affinities between PPI inhibitors and TEAD1. As a result of the virtual screening, three compounds were selected as virtual hit compounds. In order to confirm their biological activities, cellular (luciferase activity, proximity ligation assay and wound healing assay in A375 cells, qRT-PCR in HEK 293T cells) and biophysical assays (surface plasmon resonance assays) were performed. Based on the findings of the study, we propose a novel PPI inhibitor BY03 and demonstrate a profitable strategy to analyze YAP–TEAD PPI and discover novel PPI inhibitors.

## 1. Introduction

The Hippo pathway is a critical pathway for modulating organ size control and cell proliferation, differentiation and death to ensure normal tissue development [1,2,3]. Recent studies have shown that the Hippo pathway is also dysregulated in various cancers [4,5]. It functions as a suppressor pathway for the oncogenic transcription coactivator yes-associated protein (YAP), and its paralog transcriptional coactivator with the PDZ-binding motif (TAZ) [6,7]. The Hippo pathway involves various kinases, including mammalian ste20-like protein kinases (MST1/2) and large tumor suppressor kinases (LATS1/2). The final downstream step of Hippo signaling is the blocking of the transcriptional coactivators YAP and TAZ via LAS1/2-mediated phosphorylation [8]. Phosphorylation of YAP occurs at the S127 residue, thereby inducing proteasomal degradation by binding to the 14-3-3 protein [9].

Consequently, this phosphorylation prevents YAP localization from the cytoplasm to the nucleus, thus interfering with YAP binding to the transcriptional enhancer factor domain (TEAD) family of transcription factors, and blocking the expression of critical downstream target genes such as CTGF(connective tissue growth factor), CYR61(cysteine-rich angiogenic protein 61), and AMOT(angiomotin). These downstream target genes regulate cell proliferation, motility and angiogenesis, thereby indicating their potential role in cancer [10,11]. As TEAD is a key transcription factor, it has been suggested that direct inhibition of YAP–TEAD interaction by decreasing the transcriptional activity of the Hippo pathway would be an attractive strategy for cancer therapy [12,13,14,15]. Other groups have developed inhibitors that target the players upstream of Hippo signaling to induce YAP/TAZ phosphorylation by activating MST1/LATS1 kinases [16] or LATS1/2 kinases [17,18]. The strategy for targeting the most downstream molecule in the Hippo pathway is through prevention of YAP–TEAD binding to modulate transcriptional activity [19].

Verteporfin (VP) was identified as the first small molecule inhibitor of YAP that modulates the YAP-dependent transcriptional activity of TEAD [20]. However, verteporfin may not be a very promising inhibitor of YAP-TEAD owing to its poor stability and solubility, and Hippo-independent effects [21,22,23]. Some peptide-mimicking drugs, including a cyclic peptide derived from the YAP (84–100) sequence [13], a hybrid peptide from YAP74–99 and VGLL4 protein sequence [24] and, more recently, a cysteine-rich peptide TB1G2 mimicking the α-helix binding domain of YAP, have been shown to directly disrupt YAP–TEAD interactions [25].

The YAP–TEAD binding site structure has been revealed by X-ray crystal analysis, and some critical residues for the YAP–TEAD interactions have been confirmed by experimental data obtained in a previous study [26]. The YAP binding site of TEAD consists of three extensive interfaces.

Interface 1, located on the surface of TEAD, is composed of an antiparallel beta sheet, which interacts through the surface of YAP formed by a single strand. The interaction between YAP and TEAD through Interface 1 does not have a relatively strong binding interaction. Interface 2, located on the surface of TEAD, is formed with helix-turn-helix motifs and interact with an alpha-helix of YAP. In Interface 2, there is a strong attraction between the pocket formed by the helix-turn-helix motifs of TEAD and the conserved motif of YAP, LxxLF, which is a crucial hydrophobic groove. These conserved hydrophobic pockets of L65, XX, L68, and F69 binding regions can be occupied by hybrid peptides and small molecule inhibitors [24,27]. In particular, the cysteine dense peptide (CDP) TB2G1 shows exceptional examples of subnanomolar range binding affinity to TEAD through interface 2, thereby indicating that interface 2 could be druggable [25]. In addition, interface 2 plays a central role because it independently forms interactions with TEAD prior to interface 3 [28].

Interface 3 in YAP forms a three-dimensional structure similar to the omega (Ω) shape [29]; thus, it is called the Ω -loop. Interface 3 includes three hydrophobic core residues in the human YAP; these are M86, L91, and F95, which form hydrophobic interactions with TEAD at interface 3 [29]. Interface 3 is known to be a promising site for YAP-TEAD inhibitor development among the three known YAP-TEAD binding sites [13,29,30].

Taken together, targeting the N-terminal regions of YAP that are composed of various vital interfaces might be an efficient strategy to modulate YAP–TEAD protein–protein interactions. PPIs are known to be difficult to modulate with small molecules because of their vast and flat binding interface [31]. However, not all PPI sequences are critical for interactions; hot spots contribute to most of the protein-binding interactions [32]. Because the hot spot are located in a specific domain of the interfaces of a wide protein surface in the form of small binding pockets, analyzing and understanding these hot spots may provide an opportunity to identify novel potent small molecule inhibitors of YAP–TEAD PPIs that modulate transcription activity [33]. In the new drug discovery process, the role of computer-based simulation is to substantially reduce the time and cost required for new drug synthesis and biological activity tests by presenting the areas of molecules in a chemical space that can exhibit drug efficacy as precisely as possible, e.g., scaffolds. In an energy-based drug design, the energy that best represents a target system is the free energy obtained by the sum of the entropy and the energy calculated according to the 1st principle (ab initio) of a system. To obtain reliable results in biomolecular simulations, the first priority is to identify energy calculation methods that can reproduce the energy as closely as possible. To reproduce the energy and explain biological phenomena, empirical potential energy functions, called force fields, have been developed and used for biomolecular simulations that contribute to the success of energy-based computer-aided drug discovery. Because each force field is developed based on different physical phenomena and different experimental results, there are intrinsic limitations in reproducing ab initio energy. Most force fields introduce an effective pair potential approximation, where the sum of all the pair potential energies corresponds to the total energy of the system. The effective pair potential is inadequate to describe the interactions between important multibody effects, including hydrogen bonds, π–π interactions, charge transfer, induced dipole interactions, and induced-induced dipole interactions.

To overcome the disadvantages mentioned above, the Fragment Molecular Orbital (FMO) method was introduced in this work. The fragment molecular orbital method was developed by Kitaura et al. in 1999 [34]. In recent years, FMO has been increasingly used in new drug designs based on accurate energy calculations, especially in protein-ligand and protein-protein interaction analysis [35,36,37,38,39,40,41]. In order to investigate PPI, a 3-dimensional scattered pair interaction energy (FMO/3D-SPIE) analysis was introduced to sort vital interactions with the FMO method, and it efficiently correlated the results with experimental site-directed mutagenesis results [36]. In this work, we performed FMO/3D-SPIE analysis to investigate protein-protein interactions between human YAP and TEAD1 at the quantum mechanical level. We explored hotspot residues by correlating the FMO/3D-SPIE results with the site-directed mutagenesis of the YAP/TEAD complex conducted in previous studies. We focused on interface 2 because of the recent emerging interest in this YAP–TEAD binding interface [24,25,27,28]. Based on the hot spot residues, a pharmacophore model for designing inhibitors of interface 2 at YAP–TEAD1 PPIs was generated, and pharmacophore-based screening was performed by in-house libraries. Molecular docking simulations were performed to discover novel compounds that can inhibit the PPI between YAP and TEAD1. We conducted luciferase assays, proximity ligation assays(PLA), wound healing assays, TEAD target gene expression assays, and surface plasmon resonance (SPR) analysis to validate the potency of screening hit compounds [42]. In addition, we structurally minimized the BY03-TEAD1 complex using the semiparameterized FMO method and analyzed the interaction energies in the complex using the post-Hartree FMO method. Overall, the integration of the FMO/3D-SPIE and modeling simulations can provide a powerful tool for the validation of newly discovered protein–protein interaction inhibitors.

## 2. Materials and Methods

### 2.1. Protein Structure Preparation

The X-ray crystal structures of human YAP and hTEAD1 were retrieved from the Protein Data Bank (PDB ID: 3KYS). All missing side chains were filled using Prime implemented in the Maestro program [43]. Hydrogen atoms were added to the crystal structure at pH 7.0, and their positions were optimized using PROPKA implemented in the Maestro program [44]. Subsequently, restrained energy minimization was performed with an OPLS3 force field within 0.3 Å root mean square deviation [45].

### 2.2. FMO Calculations

All FMO calculations were performed using GAMESS [46]. In all FMO calculations, we used the two-body FMO method, and each residue in the protein and ligand was defined as a fragment. All input files were prepared with our in-house code in compliance with hybrid orbital projection (HOP) scheme fragmentation [47].

The two-body FMO method is composed of four steps: (1) fragmentation, (2) one fragment self-consistent field (SCF) calculation, (3) two-fragment SCF calculation, and (4) total property evaluation, the details of which have been previously described [48].

(1) In the fragmentation step, each residue in the protein, ligand, and water molecules can be defined as a fragment. Unlike the normal separation at peptide bonds in proteins, all residues in the protein are separated at the *sp3* bond between the alpha carbon and carbonyl carbon atoms in the backbone structure based on the HOP scheme [47]. The scheme was introduced to reduce the computational cost markedly and correct errors from artificial fragmentation with a projection operator [47].

(2, 3) In the second and third steps, all the molecular orbitals (MOs) on a fragment were optimized by SCF theory in the whole electrostatic field, and all electron densities were self-consistently solved through self-consistent charge iterations [35,48,49]. The difference between the second and third steps was only in the Hamiltonian operators [49]. While that the second step optimized all MOs in a fragment and included the electrostatic potential from *N-1* fragments, the third step optimized all MOs in two fragments and included the potential from *N-2* fragments.

(4) To evaluate the total properties of the entire system, all MO results obtained in the second and third steps were combined [48]. Pair interaction energies (PIEs) between two fragments were calculated, and the energy decomposition of PIEs was performed to understand the contributions of the five energy terms. The PIEs between fragments in FMO calculations were decomposed by five energy terms defined in equation 1: electrostatic $\left(\Delta E^{es}\right)$, exchange-repulsion $\left(\Delta E^{ex}\right)$, charge transfer with a higher-order mixed term $\left(\Delta E^{ct+mix}\right)$, dispersion ($\Delta E^{di})$, and solvation energy $(\Delta G_{sol}$) obtained from the polarizable continuum model (PCM).

(1) $$\Delta E^{int}\;=\Delta E^{es}+\Delta E^{ex}+\Delta E^{ct+mix}+\;\Delta E^{di}+\;\Delta G_{sol}\;\;$$

In order to investigate key protein–protein interactions between YAP and TEAD1, we analyzed the YAP/TEAD1 complex using the second-order Møller-Plesset perturbation theory (MP2) [50] and PCM [51] with a 6-31G** basis set (FMO2-MP2/6-31G**/PCM level). We then performed 3D-SPIE analysis to sort significant protein–protein interactions in the complex by selecting PIEs more stable than −3.0 kcal/mol and with a single distance of less than 5.4 Å between two fragments [36,48,52].

To obtain a more structurally stable binding mode of BY03, the energy minimization of the top-hit docking pose of BY03 was performed at the FMO-DFTB3/D/PCM level with the third-order corrected density functional tight-binding (DFTB3) method using the 3OB parameter set [53,54], UFF-type dispersion correction (D) [55], and polarizable continuum model [56]. In energy minimization, the residues within 10.4 Å of the ligand were included and fixed, while only the ligand was fully flexible. The energy minimization calculations of BY01 converged in 72 steps, those of BY02 converged in 80 steps, and those of BY03 converged in 153 steps. To analyze the interactions at the molecular level between the TEAD1 and PPI inhibitors, we performed FMO calculations with energy-minimized ligand and full protein complexes at the FMO2-MP2/6-31G**/PCM level. The binding affinity of the protein–ligand interactions was approximated as the sum of the PIEs. All residues in the crystal structure were included in the energy decomposition analysis.

### 2.3. Virtual Screening

To identify compounds that inhibit the protein–protein interactions between TEAD1 and YAP, pharmacophore-based virtual screening was carried out using 7.0 million compounds of an in-house synthetic database with the phase implemented in the Schrödinger suite [57]. The pharmacophore was generated with the PPI between TEAD1 and YAP. The top-hit inhibitors, BY01, BY02 and BY03 were selected for pharmacophore-based screening.

### 2.4. Molecular Docking

Molecular docking was conducted to investigate the interactions between BY01, BY02, BY03 and TEAD1 (PDB:3KYS) using Glide in the Schrödinger suite [57].The docking pose of compounds BY01, BY02 and BY03 in complex with TEAD1 was selected using glide score, e-model score, and visual inspection.

### 2.5. SPR Assay

SPR analysis was used to investigate whether BY03 binds to TEAD1. BY03 changed the refractive index and mass concentration when injected into the TEAD1-immobilized sensor surface. To confirm the binding selectivity of BY03 compounds for TEAD1, we performed SPR analysis of binding between TEAD1 and BY03.

The purified full-length TEAD1 protein with a His-tag was prepared by Young Frontier Co., Ltd. The interaction between BY03 and TEAD1 was investigated using the ProteOnTMXPR36 Protein Interaction Array System (Bio-Rad Laboratories, Hercules, CA, USA). Purified TEAD1 was immobilized by amine coupling on a ProteOn GLH sensor chip. The ligand was diluted with 1× phosphate-buffered saline with Tween 20 and 1% DMSO at different concentrations (0, 6.25, 12.5, 25, and 50 μM), and then passed over the chip at a flow rate of 100 μL/min. The data were analyzed by ProteOn Manager Software 2.0 (Bio-Rad Laboratories, Hercules, CA, USA) using standard Langmuir models to fit the kinetic data. The association constant (k_a_ in M/s) represents the rate of complex formation, whereas the dissociation constant (k_d_ in s^−1^) represents the rate of complex decay. High-affinity interactions were characterized by low K_D_ values, whereas rapid recognition and binding of the interactants (rapid “on rate,” or high k_a_) and stability of the complex formed (slow “off rate,” or low k_d_) were represented by the equation K_D_ = k_d_/k_a_.

### 2.6. Cell Culture and Stable Cell Lines

Stable cell lines, namely, HEK293T, A375, and A375 TEAD luciferase cells, were maintained in DMEM (Dulbecco’s Modified Eagle Medium) containing 10% fetal bovine serum, penicillin, and streptomycin at 37 °C in a humidified 5% CO_2_ incubator. For the construction of A375-TEAD luciferase stable cell line, A375 cells were infected with TEAD luciferase reporter lentivirus using polybrene (Millipore, Burlington, MA, USA), and then selected with puromycin.

### 2.7. Luciferase Reporter Assay

HEK293T cells were seeded in 24-well plates (1 × 10^5^ cells/well) 1 d before transfection. A mixture of 8xGTIIC TEAD promoter-luciferase reporter, pGL-8xGTIIC-firefly, and pRL-CMV-Renilla were cotransfected into HEK293T cells. After 6 h, cells were treated with 0.01, 0.1, 1, 10, or 100 μM inhibitors for another 24 h. Cells were lysed, and luciferase activity was measured using a dual-Glo Luciferase Assay (#E2920; Promega, Madison, WI, USA) according to the manufacturer’s instructions. Luciferase activity was measured using a VICTOR Nivo™ Multimode Microplate Reader (PerkinElmer, Waltham, MA, USA). Transfection efficiency was normalized to the CMV immediate early enhancer/promoter region (pRL-CMV) activity as an internal control. A375 TEAD luciferase cells were seeded in 96-well plates (4 × 10^4^ cells/well) 1 d before the minor molecule treatment. After 24 h, cells were treated with 0.01, 0.1, 1, 3, or 10 μM small molecules for another 24 h. Cells were lysed, and luciferase activity was measured using an ONE-Step™ Luciferase Assay System (#60690-1; BPS Bioscience) according to the manufacturer’s instructions. Luciferase activity was measured using a VICTOR Nivo™ Multimode Microplate Reader (PerkinElmer).

### 2.8. mRNA Expression

For quantitative real-time PCR, total RNA was isolated using RNeasy Mini Kit (Qiagen, Hilden, Germany) according to the manufacturer’s protocol. cDNA was synthesized from total RNA using iScript™ Reverse Transcription (BIO-RAD) with the unique blend of oligo(dT) and random hexamer primers. Real-time PCR was carried out by the QuantStudio™ 3 Real-Time PCR System with PowerUp™ SYBR™ Green Master Mix (Applied Biosystem, Waltham, MA, USA).

PCR products were designed to amplify specific mRNA fragments and as a displayed unique dissociation (melting) curve. PCR conditions were 95 °C (10 min) and 40 cycles at 95 °C (15 s) and 60 °C (1 min). The threshold cycle (Ct) value for each gene was normalized to the Ct value for Actin. Relative mRNA expression was calculated using the ΔΔCt method. Primers used for human cell lines: Cyr61, 5′-AGCCTCGCATCCTATACAACC -3′ and 5′- TTCTTTCACAAGGCGGCACTC -3′; Amot, 5′- CAGCTTGCAGAGAAGGAATATGAG -3′ and 5′- CTGGCTTTCTTTATTTTTTGCAAAG -3′; Actin, 5′ -GCCGACAGGATGCAGAAGGAGATCA -3′ and 5′ -AAGCATTTGCGGTGGACGATGGA -3′.

### 2.9. Proximity Ligation Assay (PLA)

A375 cells were seeded in 24-well plates on coverslips with Poly-L-ornithine solution (Sigma, P4957, St.Louis, MO, USA) diluted 1:20 at 37 °C for 30 min with a quick phosphate-buffered saline (PBS) wash prior to cell seeding. The following primary antibody was used for PLA assay: YAP (63.7; Santa Cruz Biotechnology), TEAD (D3F7L, Cell signaling). Cells were then subjected to proximity ligation assays using the Duolink™ In Situ Red Starter Kit Mouse/Rabbit (DUO92101; Sigma-Aldrich/Merck) and following the manufacturer’s instructions [58].

## 3. Results

### 3.1. FMO Study of the YAP-TEAD1 Complex

#### 3.1.1. Analysis of the YAP-TEAD1 Interaction at FMO Study on Interface 2 with FMO Calculation

**Figure 1:**
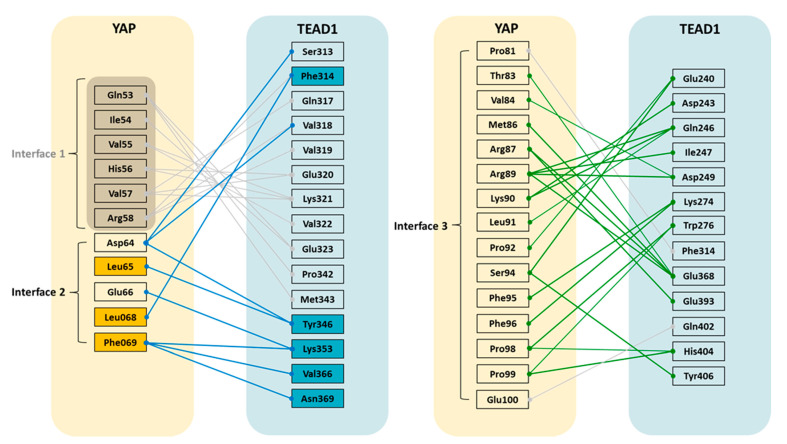
PPIs map of YAP-TEAD1. The 3D-SPIE-based interaction maps consist of interface 2 (**left**) and interface 3 (**right**) YAP–TEAD1 PPIs. The main interactions in the interface 2 of YAP-TEAD1 are shown by blue lines (**left**). The orange box represents the LxxLF motif in YAP, and the blue box represents binding partner residues in TEAD1 for the YAP LxxLF motif. The main interactions in interface 3 are represented by green lines (**right**). Left gray lines indicate interface 1, and right gray lines indicate amino acids excluded from interface 3. All interactions (shown in this map) whose magnitudes are ignored have attractive PIE values more stable than −3.0 kcal/mol.

**Figure 2:**
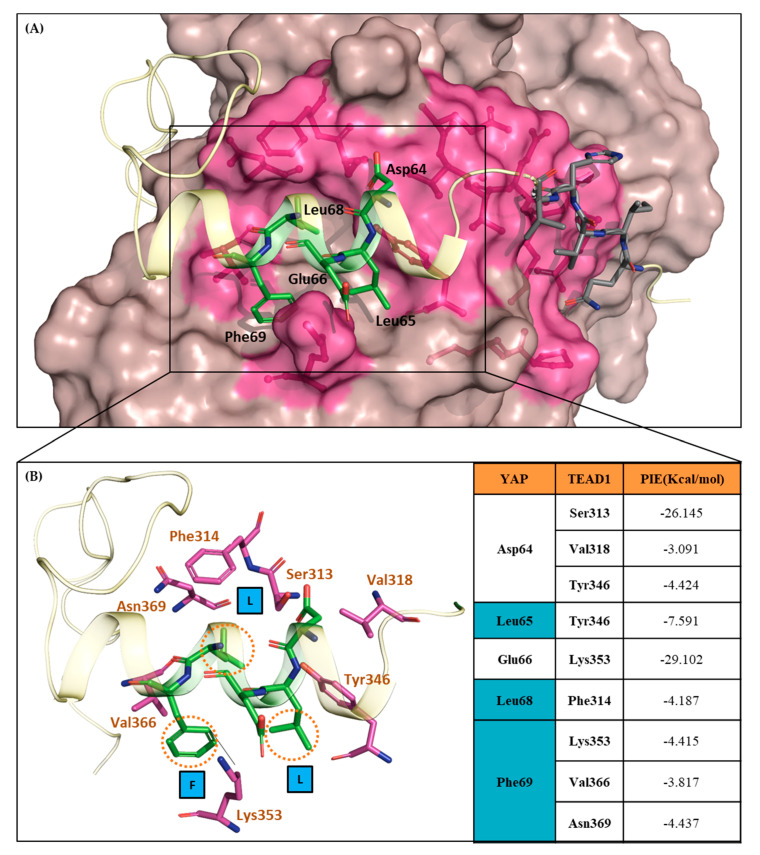
FMO analysis for interface 2 of YAP–TEAD1 PPIs. (**A**) Hot spot residues of YAP, namely, Asp64, Leu65, Glu66, Leu68, and Phe69, in interface 2 are shown as green sticks, Yellow indicates YAP alpha-helix and dark pink TEAD, and Raspberry color represents the TEAD1 interaction domain for YAP hot spot residues in interface 2. (**B**) TEAD1 hot spot residues, namely, Ser313, Val318, Tyr346, Lys353, Phe314, Val366, and Asn369, are shown as magenta sticks. The blue box indicates the LxxLF hydrophobic motif in YAP. The detailed PIEs are described in the right panel.

Interface 2 of YAP–TEAD1 PPIs comprises highly conserved hydrophobic pockets that possess an LxxLF motif in YAP. The TEAD1 binding site of interface2 comprises a helix-turn-helix motif which contains a variety of hydrophobic residues. These hydrophobic pockets have been considered important binding sites for PPIs in recent studies [25,27]. FMO calculation of the part of the YAP-TEAD1 complex which is contacted through the interface 2 was performed. Preferentially, we confirmed 11 strong binding interactions near interface 2 of YAP: Gln53, Ile54, Val55, His56, Val057, Arg058, Asp64, Leu65, Glu66, Leu68, and Phe69. As Interfaces 2 and 1 are closely linked, the interaction regions specific to interface 2 only included Asp064, Leu65, Glu66, Leu68, and Phe69. These YAP sequences have noticeable contact with TEAD1 at Ser313, Phe314, Val318, Tyr346, Lys353, Val366, and Asn369, with PIEs more stable than −3 kcal/mol as summarized in Table S1, and Figure 1 and Figure 2. These results agree with previous mutagenesis studies, which proved the critical role of YAP interface 2 residues, namely Asp64, Leu65, Leu68, and Phe69, in regulating TEAD1 binding affinities [26,28,59]. Although Arg58 was also close to interface 2, it was located on interface 1 and seemed to contribute to the formation of YAP intra-interactions with Asp64 using strong electrostatic interactions with −64.558 kcal/mol of energy. The most crucial hydrophobic moiety in interface 2 consisted of Leu65, Leu68, and Phe69. Hydrophobicity of these residues in interactions was described by dispersion energy term(∆E^di^) in PIEDA with −5.478, −4.061, and −8.318 kcal/mol (summation of ∆E^di^ for TEAD1 binding residues for YAP Phe69; Lys353, Val366, and Asn369), respectively (Table S1).

Notably, Phe69 mainly formed hydrophobic interactions with Val366 and Lys353, which form a hydrophobic moiety in the LxxLF motif. Although Lys353 included a positive guanidine group, Phe69 created hydrophobic interactions with Lys353 and Val366, thereby constructing hydrophobic pockets. The FMO results also detected strong interactions between YAP/Asp64 and TEAD1. YAP/Asp64 formed three interactions with TEAD1/Ser313, Val318, and Tyr346. In particular, Asp64 interacted more strongly with TEAD1/Ser313 than others with a PIE of −26.145 kcal/mol, which is predominantly composed of electrostatic interactions implied by ∆E^es^ terms in PIEDA (Table S1). This might explain the importance of the hydrogen bonds between YAP/Asp64 and TEAD1/Ser313. In addition, TEAD1/Tyr346 formed a hydrogen bond with YAP/Asp64 with an energy of −4.424 kcal/mol. These results demonstrate that maintaining hydrogen bonds in YAP Asp64 to TEAD1 is essential for stable binding. Conversely, YAP/Glu66 formed a favorable electrostatic binding interaction with TEAD1/Lys353 with an energy of −29.102 kcal/mol, which has strong ∆E^es^ terms in PIEDA.

#### 3.1.2. Analysis of the YAP-TEAD1 Interaction at Interface 3 with FMO Calculation

Interface 3 is the most crucial of the three interfaces involved in the YAP–TEAD1 PPIs [26,29,30]. In Interface 3, owing to the strong binding affinity of YAP-TEAD1 complex, some peptidomimetic PPI inhibitors developed targeting interface 3 [60,61].

**Figure 3:**
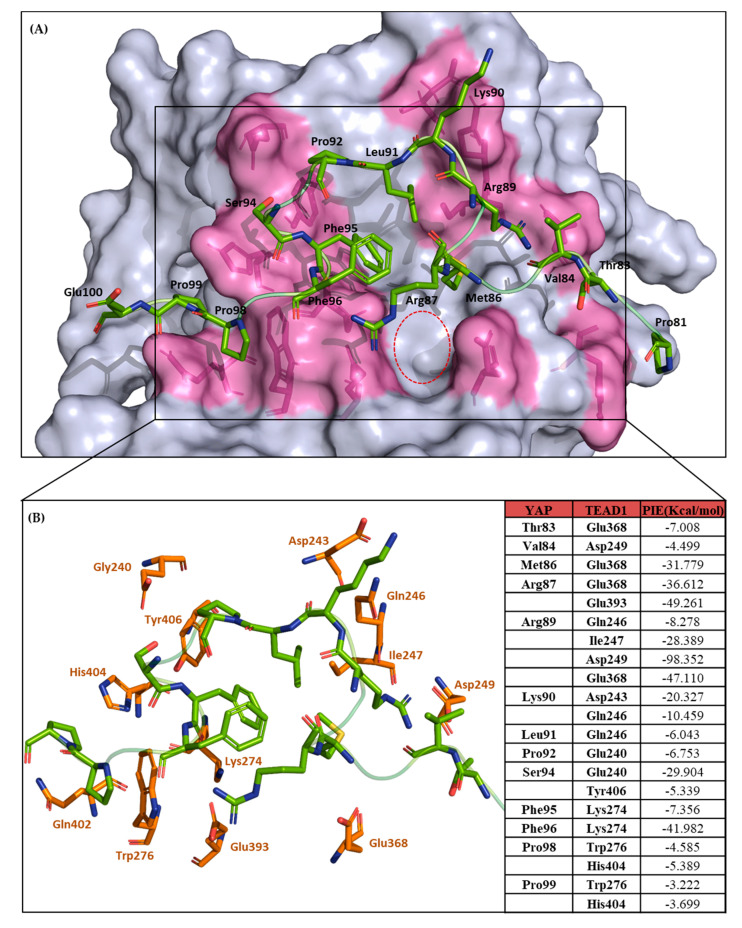
FMO analysis for interface 3 of YAP–TEAD1 PPIs. (**A**) Hot spot residues of YAP, namely, Thr83, Val84, Met86, Arg87, Arg89, Lys90, Leu91, Pro92, Ser94, Phe95, Phe96, Pro98, and Pro99, are shown as green sticks. The green loop indicates YAP, the light greyish-blue TEAD1, and raspberry color indicates the TEAD1 interaction domain for YAP hot spot residues in interface 3. (**B**) TEAD1 hot spot residues in interface 3 are shown as orange sticks. Details of PIEs are described in the right panel.

Since the surface of the interface 3 of TEAD1 is wide and long, to illuminate the interactions between the two proteins at interface 3, as shown in Figure 3, the peptide with 20 amino acids belonging to the interface 3 of YAP (Pro81~Glu100) was introduced for the FMO calculation. According to the PIEDA analysis, among the 20 amino acids, 15 amino acids, Pro81, Thr83, Val84, Met86, Arg87, Arg89, Lys90, Leu91, Pro92, Ser94, Phe95, Phe96, Pro98, Pro99 and Glu100, are attractive with the amino acids in TEAD1 interface 3, as summarized in Table S2, and Figure 1 and Figure 3. Especially, five amino acids, Arg87, Arg89, Lys90, Ser94, and Phe96, mainly contribute to the total e binding energy of the complex. The electrostatic attraction energy corresponds to the major portion of the binding energy of the complex. YAP-Arg87 strongly attracts TEAD1-Glu368 and Glu393, which form a shallow binding pocket, shown as red dashed circle in Figure 3. Arg89 showed the highest number of potent interactions with TEAD1. It interacted with Gln246 with an energy of −8.278 kcal/mol, Ile247 at −28.389 kcal/mol, Asp249 at −98.352 kcal/mol, and Glu368 at −47.110 kcal/mol. Interestingly, high ∆E^es^ in PIEDA revealed that Arg89 interacted with Asp249 primarily through strong electrostatic interactions, and also possessed hydrogen bonds (Table S2). Otherwise, Met86, Lys90, Ser94, Phe95, and Pro98 showed hydrophobicity in PIE by ∆E^di^ terms in PIEDA. Lys90 mainly showed electrostatic interactions with Asp243 of TEAD1 with −20.327 kcal/mol. In addition, Lys90 interacted with Gln246 of TEAD1 with an electrostatic force of −10.459 kcal/mol, thereby forming a hydrogen bond and hydrophobic contact, which are described by the ∆E^di^ and ∆E^es^ terms in PIEDA (Table S2).

Met86 possesses strong interactions of −31.779 kcal/mol PIE composing of hydrophobic and electrostatic terms described by ∆E^di^ and ∆E^es^ in PIEDA. In addition, Met86, located in a binding pocket at interface 3 with Phe95, might be an essential residue for the formation of stable YAP–TEAD1 PPIs. Along with Met86, Phe95 is one of the few amino acids embedded in the shallow binding pocket at interface 3 of TEAD1 [62]. It retained hydrophobicity-driven interactions with Lys274 for TEAD1 with electrostatic forces of −7.356 kcal/mol. Moreover, as described above, YAP Ser94, Phe96, Arg89, and Phe95 have been revealed as critical binding residues in YAP–TEAD1 PPIs by previous mutagenesis studies [26,29,30,59,63,64]. Phe96 interacts with Lys274 via electrostatic interactions. In addition, it constructs intra-interactions that stabilize YAP conformation by interacting with cation-pi interactions with Arg87. Intra-interactions between Arg87 and Phe96 were described with a PIE of −9.197 kcal/mol (data not shown) [13]. Interface 3 contains more amino acid contacts than interface 2, mainly composed of an electrostatic term.

### 3.2. Hot Spot-Focused Virtual Screening

**Figure 4:**
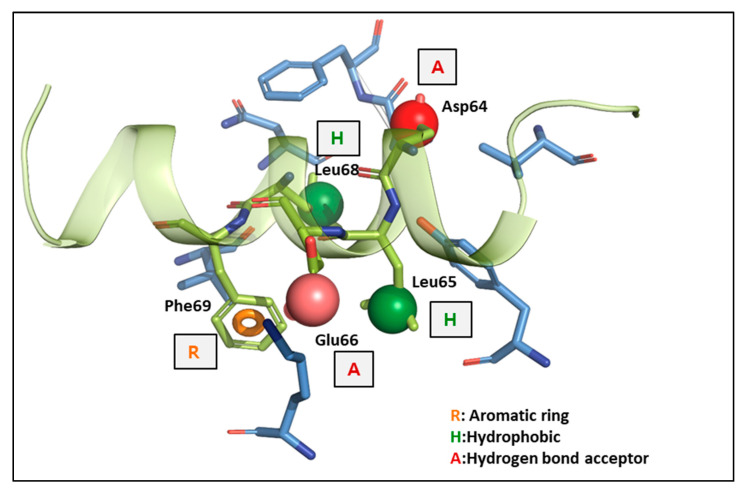
Pharmacophore models derived from the hot spot at interface 2 of YAP–TEAD PPIs. R, aromatic ring; H, hydrophobic; A, hydrogen bond acceptor. The green helix and sticks represent the YAP region, and the light blue stick represents the TEAD1 region.

**Scheme 1:**
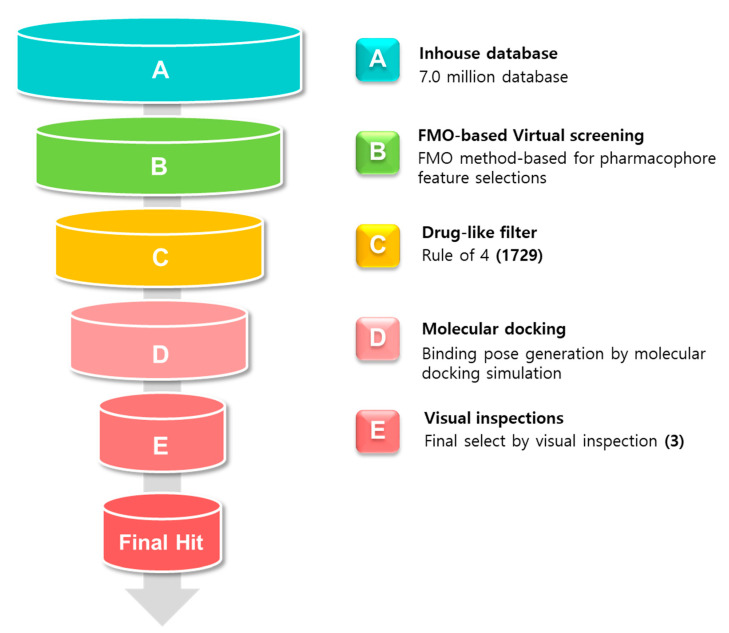
Virtual screening process for the screening of the YAP-TEAD1 inhibitor.

Comprehensive analysis of previous studies on the structure and interaction of the Yap-TEAD1 complex concluded that interface 2 is a suitable site for the development of Yap-TEAD1 interaction inhibitors [24,25,27,28]. Therefore, an FMO-based pharmacophore (Figure 4) was constructed for interface 2 with the results summarized in Table S1 and Figure 2, and molecular docking-based virtual screening was performed with the pharmacophores. A flowchart of the virtual screening process for YAP–TEAD1 PPI inhibitors targeting interface 2 is shown in Scheme 1. The virtual screening was performed using an in-house virtual library, which is a modification of the ZINC library (7 million virtual compounds) to facilitate our procedure proposed in the Scheme 1.

Pharmacophore models were generated from previously identified hot spot residues of YAP at interface 2. In the pharmacophore model of interface 2, we identified five pharmacophore features composing of two hydrogen bond acceptors, two hydrophobic and one aromatic ring, that are essential for hydrophobic and pi–cation interactions. We selected pharmacophore features of these residues by referring to the energy terms of PIEDA: Asp64 as a hydrogen acceptor, Leu65 as hydrophobic, Glu66 as a hydrogen acceptor, Leu68 as hydrophobic, and Phe69 as hydrophobic and aromatic ring. The details of interface 2 pharmacophores are shown in Figure 4.

To consider drug likeliness for PPI inhibitors, the Rule-of-4 (RO4) filter was introduced: molecular weight (MW) > 400 Da, AlogP > 4, the number of rings > 4, and the number of hydrogen bond acceptors > 4, which replaced Lipinski’s rules for considering the hydrophobic nature of PPIs [65].

**Figure 5:**
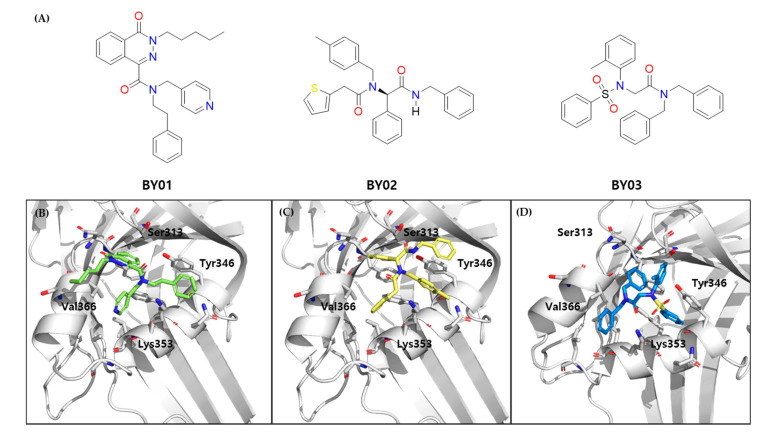
Structure of the three selected virtual hits from FMO-based virtual screening. (**A**) The structure of BY01, BY02, BY03. B. Docking poses in the TEAD1 binding pocket of BY01, (**B**); BY02, (**C**) and BY03, (**D**) using Glide in the Schrödinger suite.

However, the face 2 pharmacophore did not satisfy some of the criteria of the RO4, and the RO4 criteria were flexibly applied in the PPI drug likeness filtering; for example, the number of hydrogen bond acceptor cut off set was set as greater than three. As a result of examining the binding pose of the screening result, we concluded that including four aromatic rings tended to have a stable binding pose for interface 2. A total of 1729 compounds were screened using FMO-based pharmacophore virtual screening and RO4 filters. Molecular docking was performed to generate the binding positions of the compounds. After visual inspections for selecting molecules, we finally concluded the three candidates to be BY01, BY02, BY03, as shown in Figure 5.

### 3.3. Luciferase Report Assay for Ligands

**Figure 6:**
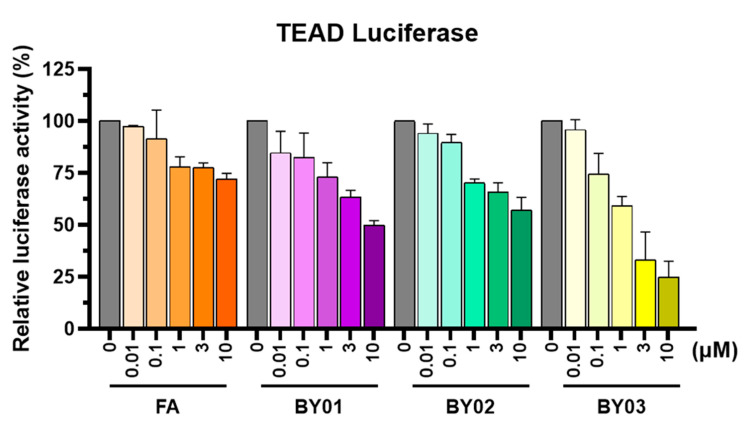
TEAD reporter luciferase activity observed in A375 cells treated with 10 nM to 10 μM of hit compounds with FA (flufenamic acid) 24 h post-transfection (*n* = 3).

**Figure 7:**
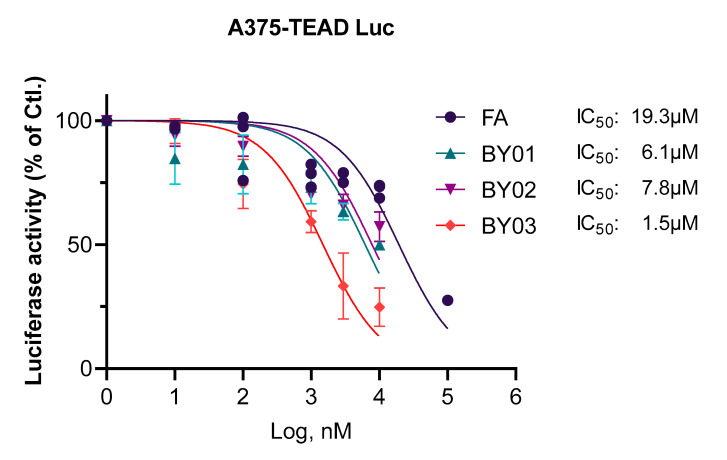
TEAD luciferase activity inhibition observed in A375 cells treated with hit compounds and FA (flufenamic acid) 24 h post-transfection (*n* = 3).

YAP–TEAD PPI inhibition was confirmed by TEAD-dependent luciferase by overexpressing the TEAD luciferase plasmid in HEK293T, the human embryonic kidney cells used in the previous test. Luciferase expression level decreased up to 68% in the presence of BY03 at 20 μM with ectopic expression of YAP-S127A, with phosphorylation by LATS 1/2 in a Hippo pathway-dependent manner (Figure S1). The same results, with the tendency to decrease the luciferase activity, were obtained when experiments to confirm YAP–TEAD PPI inhibition in cancer cells were performed in A375, which consisted of melanoma cancer cells with increased YAP activity. We measured TEAD transcriptional activity in A375 TEAD luciferase-stable melanoma cells in the presence of our compounds using a TEAD reporter construct. An 8X-GTIIC (Repeat 8 Times TEAD binding motif in the promoter region) luciferase reporter was placed downstream of the TEAD sites; therefore, the expression level of luciferase represented TEAD transcriptional activity. As shown in Figure 6, a significant decrease of up to 75% in luciferase expression was observed in the presence of BY03 at 10 nM to 10 μM with an IC50 value of 1.5 µM. BY01 and BY02 also showed a decrease in the luciferase expression up to 50%, with 30% in the presence of BY01,02 at 10 nM to 10 μM with an IC50 value of 6.1 μM to 7.8 μM, respectively, whereas the IC50 value of the positive control compound flufenamic acid was obtained as 19.3 µM (Figure 7).

### 3.4. SPR Assay for Ligands

SPR analysis was performed to confirm whether the selected compounds directly bind to TEAD1. Injected hit compounds altered the mass concentration and refractive index when injected into the YAP and TEAD1-immobilized sensor surfaces.

**Figure 8:**
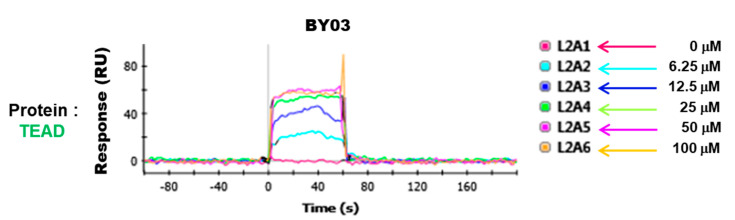
SPR analysis of BY03 to TEAD1. Sensorgrams of BY03 binding to the TEAD1. In the SPR experiment, TEAD1 was immobilized onto the vertical channels of a NeutrAvidin-precoated ProteOn GLH sensor chip, and BY03 was injected into the horizontal channels of the sensor chip at different concentrations of 0, 6.25, 12.5, 25, 50, and 100 μM.

The BY03 compounds exhibited the most potent dose-dependent change, with a K_D_ value of 9.4 μM for TEAD1. We could not obtain proper sensorgram data or K_D_ values to indicate the binding affinity of BY01 and BY02. Overall, SPR assays suggest that BY03 can directly bind to TEAD1 more efficiently than BY01 and BY02 (Figure 8).

### 3.5. YAP-TEAD Interaction for the Proximity Ligation Assay

**Figure 9:**
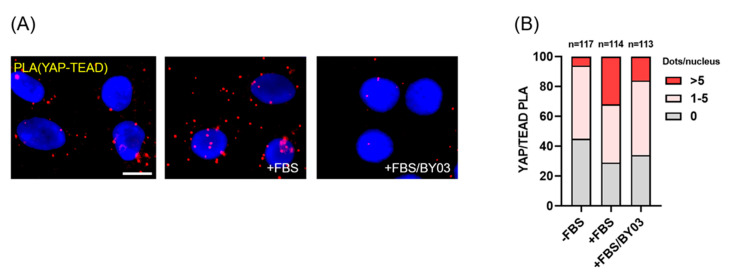
Fluorescence signals generated by proximity ligation assay demonstrate that endogenous TEAD and YAP are closely located in the nucleus at MSTO-211H. (**A**) Representative fluorescence images of nuclear YAP-TEAD PLA signals from assay (Scale bars, 10 μm). (**B**) Stick graph presentation by % of cells with corresponding PLA signal ranges per nucleus.

To elucidate the Yap-TEAD complex inhibition by BY03 treatment, the interaction between endogenous YAP and TEAD was analyzed by a proximity ligation assay (PLAs), with specific antibody-based signal of protein-protein interactions in close proximity. Interestingly, quantification of nucleus PLA signals were attenuated by the BY03 treatment (Figure 9).

### 3.6. TEAD Target Gene Expression

**Figure 10:**
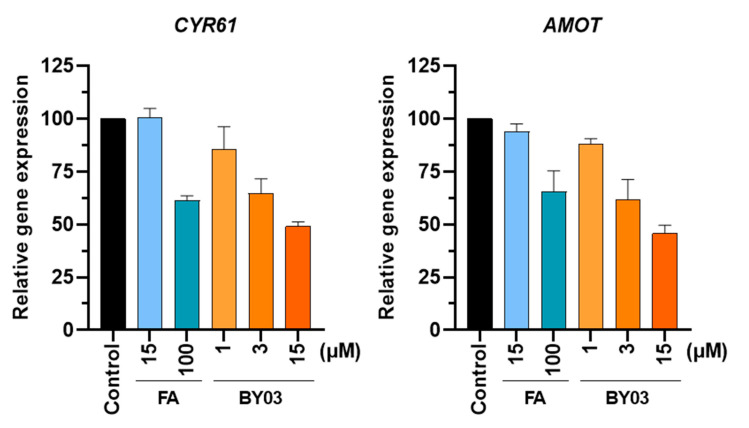
Expression of YAP-TEAD target genes (CYR61 and AMOT) in HEK293T cells incubated in the presence of BY03 at 1 μM, 3 μM, 15 μM, respectively for 24 h. Flufenamic acid (FA) was treated with 15 μM and 100 μM, respectively. mRNAs were measured by qRT-PCR (*n* = 3).

We tested the effect of BY03 on YAP-TEAD target genes. We analyzed the expression mRNAs of the target genes (CYR61, AMOT) by quantitative-real time PCR (qRT-PCR) (Figure 10). BY03 caused a decrease of 50% and 55% for CYR61, AMOT mRNAs expression, respectively. Flufenamic acid caused no noticeable reduction of mRNAs expression in the same dose at 15 μM.

### 3.7. FMO Analysis of Specific Interactions between BY03 and TEAD1

**Figure 11:**
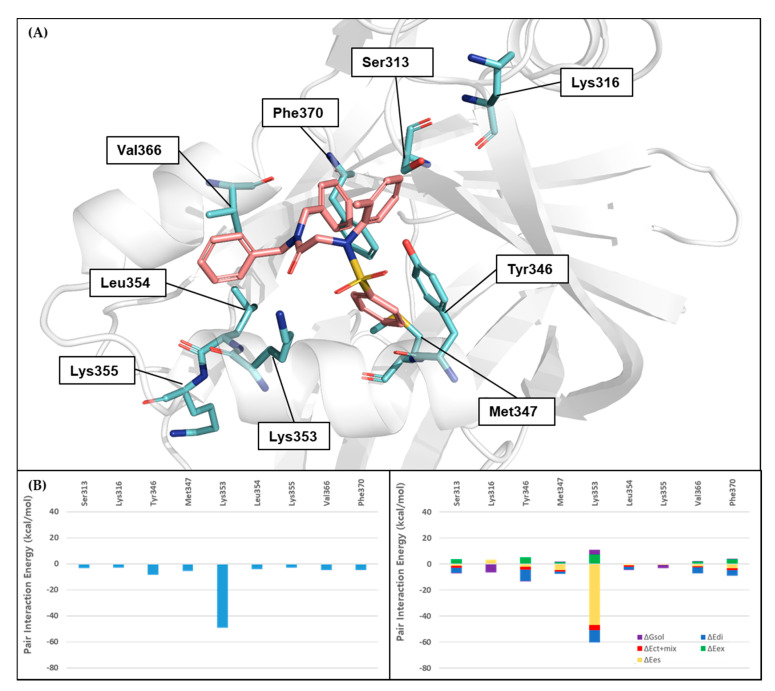
FMO results for BY03 in complex with interface 2 in TEAD1. PIEDA results of BY01 and BY02 are shown in Table S4 and S5, respectively. (**A**) The structure of BY03 binding to the interface 2 of TEAD1. The ligand is shown in light pink, the protein residues are shown in cyan, and the nitrogen and oxygen atoms are shown in blue and red, respectively. (**B**) The left bar plot describes the PIEs of the significant residues in the interface2 of TEAD1, while the right bar plot describes the PIEDA of these critical interactions. The electrostatic, exchange repulsion, charge transfer with higher order mixed term, dispersion, and solvation energy terms are shown in yellow, green, red, blue and purple, respectively.

In order to analyze the binding interactions between TEAD1 and BY03 at the molecular level, we performed FMO calculations on the TEAD1/BY03 complex at the FMO-MP2/6-31G**/PCM level after we structurally minimized the complex at the FMO-DFTB3/D/PCM level. BY03 interacted with TEAD1 with a PIE of −86.431 kcal/mol. The BY03 interaction residues in TEAD1 were Ser13, Lys316, Tyr346, Met347, Lys353, Leu354, Lys355, Val366, and Phe370, as shown in Figure 11 and Table S3. Some of these residues have been mentioned in previous hot spot analyses for interface 2 of the YAP-TEAD1 PPIs. Lys353 had the strongest interaction with BY03 with a PIE of −49.137 kcal/mol. This interaction was mainly driven by electrostatics as indicated by the ∆E^es^ term in PIEDA; it formed a pi-cation bond with benzene in BY03 and a hydrogen bond with oxygen in benzene sulfonamide in BY03. Tyr346 contacted BY03 with a PIE of −8.346 kcal/mol, which was mainly driven by hydrophobicity as indicated by the ∆E^di^ terms in PIEDA.

**Figure 12:**
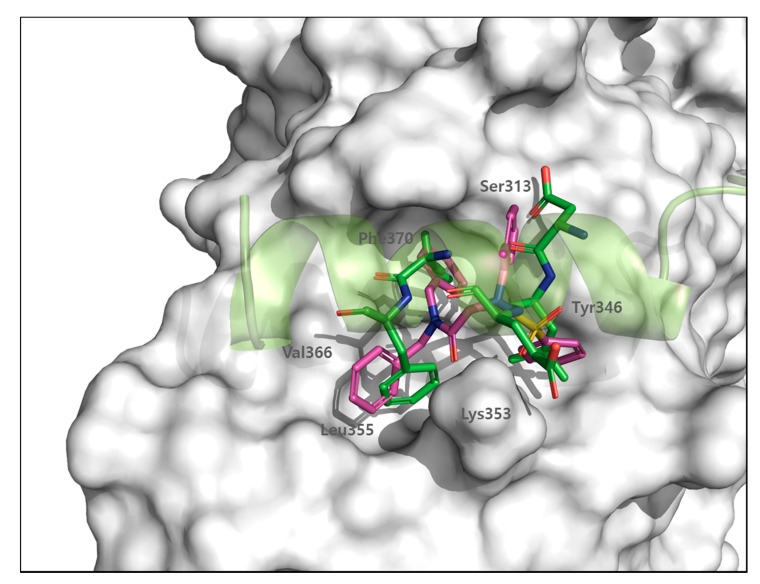
Overlay structure of BY03 to YAP at interface 2. The pink color represents BY03 and the green color represents YAP. Grey letters represent TEAD1 binding residues in interface 2 for BY03.

The BY03 scaffold could satisfy the fundamental interactions of interface 2 by substituting the positions of Phe69, Leu65, and Glu66 in the LxxLF motif, which are crucial for YAP–TEAD1 PPI, as shown in Figure 12. The benzene group of BY03 replaced Phe69 in YAP on interaction with Lys353 of TEAD1. It maintained good hydrophobicity for binding pockets and seems to construct pi–cation interactions with Lys353.

The other benzene in group BY03 replaced the Leu68 binding moiety in YAP, thereby forming hydrophobic interactions with Phe314 in TEAD1. In addition, the oxygen of sulfonamide in BY03 replaced the YAP Glu66 hydrogen bond with TEAD1. The importance of these YAP interactions was revealed from a previous FMO/3D-SPIE analysis of YAP–TEAD PPIs, and other studies [28,59,63].

In addition, toluene linked to sulfonamide in BY03 caused structural interference for the positions of YAP/Asp64 binding for TEAD1/Ser313. It might have been more potent if BY03 satisfied the interaction features between YAP/Asp64 and TEAD1/Ser313. However, the binding moiety was quite far from the other features because BY03 was too small to cover all pharmacophore features. Overall, BY03 had strong interactions that satisfied the critical interactions of YAP-TEAD1 PPIs at interface 2.

## 4. Discussion

The FMO method provides detailed interaction information on protein-protein complexes and protein-ligand complexes. In this study, the PPIs of YAP-TEAD1 were quantitatively analyzed using the FMO method at a correlated MP2/6-31G**/PCM level.

In addition, the FMO/PIEDA method provided insights into identifying the contribution of different energy components from PIEs, which means that the FMO method could provide crucial information for nonbonding interactions. To understand hydrophobicity, hydrogen bonds, salt bridges, and induced charges, which are common interactions in protein interaction systems, can be elucidated at the molecular level by this method.

We demonstrated how PPI hot spots were identified by the FMO method and applied them to discover novel PPI inhibitors through structure-based virtual screening. By utilizing the identified hot spot information from the FMO results, we developed a novel structure inhibitor, BY03, for TEAD1. The activity of BY03 was confirmed by luciferase reporter assay with a significant decrease in luciferase expression by an IC50 value of 1.5 µM. BY03 decreased mRNA expression of the TEAD target genes, CYR61 and AMOT by 50% and 55% respectively. The binding affinity of BY03 to TEAD1 was confirmed by SPR assays with a K_D_ value of 9.4 μM. The Proximity ligase assay result confirmed the inhibitory effect of BY03 to YAP-TEAD interactions. Finally, we conducted a wound healing assay to confirm phenotypic changes in cancer cells (Figure S2).

The results of this study can be used to develop novel PPI inhibitors and conduct a detailed analysis of the mode of action of PPI inhibitors using the FMO method. While the FMO method was used to make a pharmacophore model and perform binding mode analysis, its applications to molecular docking and quantitative binding affinity prediction are promising [66,67]. Therefore, the FMO method is an attractive tool for analyzing the molecular interactions between ligands and proteins and developing novel inhibitors for PPIs in drug discovery.

## 5. Conclusions

The FMO method can provide detailed information on protein-protein complexes with the FMO/3D-SPIE method. The detailed information was used to find hot spots and incorporated these in structure-based virtual screening for identifying PPI inhibitors between YAP and TEAD1. Therefore, the FMO method is an effective tool to integrate molecular interactions from ligand-protein interactions to protein-protein interactions for developing novel inhibitors for PPIs in structure-based drug discovery.

## Data Availability

Data is contained within the article or supplementary material.

## Supplementary Materials

The following are available online at https://www.mdpi.com/article/10.3390/cancers13164246/s1. Figure S1: TEAD reporter luciferase activity observed in HEK293T cells treated with flufenamic acid and BY03., Figure S2: BY03 attenuates cancer cell migration, Table S1: PIEDA of YAP/TEAD1-IF2 complex (PDB ID: 3KYS). Table S2: PIEDA of YAP/TEAD1-IF3 complex (PDB ID: 3KYS). Table S3: PIEDA of BY03 and TEAD1 complex (PDB ID: 3KYS). Table S4: PIEDA of BY01 and TEAD1 complex. Table S5: PIEDA of BY02 and TEAD1 complex.
