# Supplementary material for: Hot Spot Analysis of YAP-TEAD Protein-Protein Interaction Using the Fragment Molecular Orbital Method and Its Application for Inhibitor Discovery
Source: Cancers (Basel). 2021 Aug 23;13(16):4246. doi: 10.3390/cancers13164246 (PMC8391968; doi:10.3390/cancers13164246)
Supplement: Supplementary file 1 [file cancers-13-04246-s001.zip › cancers-1354302-supplementary.pdf]

cancers

Supplementary Material: Hot Spot Analysis of YAP-TEAD Protein-Protein Interaction Using the Fragment Molecular Orbital Method and Its Application for Inhibitor Discovery

Jongwan Kim, Hocheol Lim, Sungho Moon, Seon-Yeon Cho, Min Hye Kim, Jae Hyung Park, Hyun Woo Park and Kyoung Tai No

Supplement Methods

Wound healing assay

A375 cells were incubated in 24-well plates and cultured until full confluency. Cells were scratched by 200-μl pipette tip and then washed with media to remove cell debris, and the media was replaced. Photographs of the wound region were taken at 0 and 48 h. Cell free area was measured using TScratch software [1].

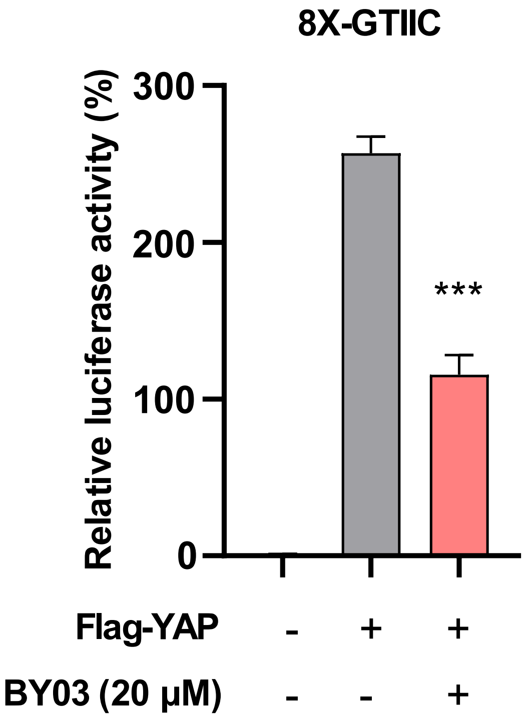

Figure S1. BY03 negative regulates YAP-dependent TEAD activity. TEAD reporter luciferase activity observed in HEK293T cells treated with 20 μM of BY03 with overexpression of YAP-S127A at 24 h post-transfection.

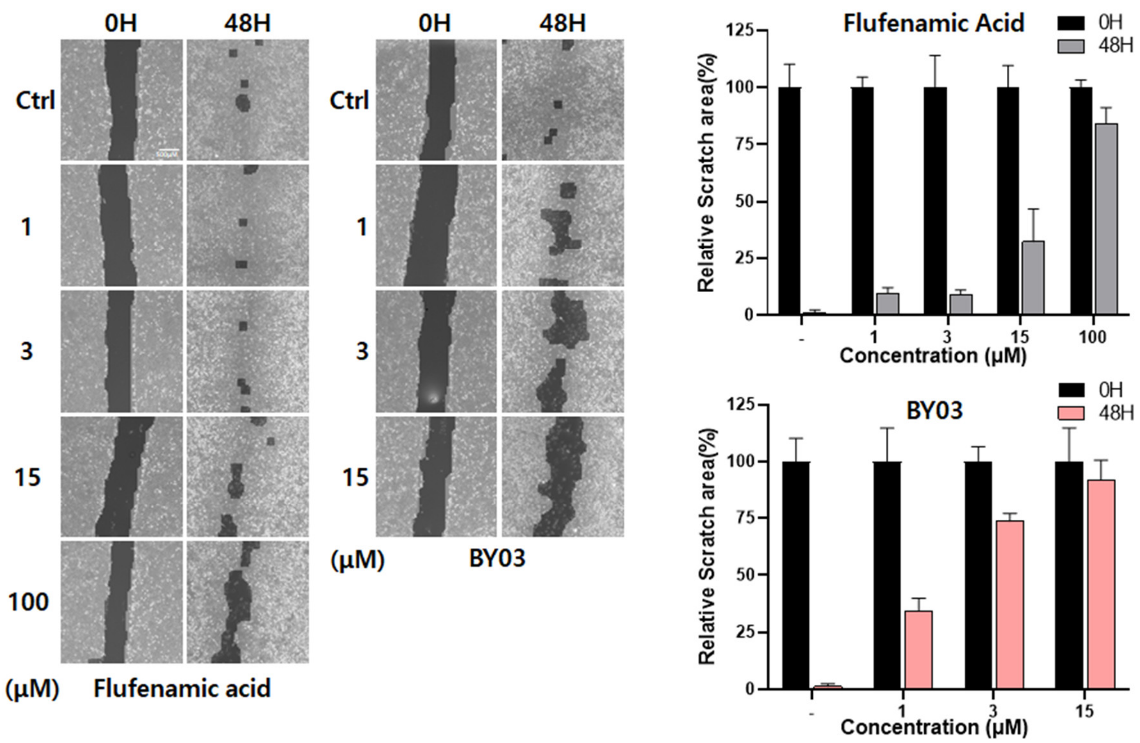

Figure S2. BY03 attenuates cancer cell migration. Wound-healing assay for migration of FA or BY03 treatment and the wounded areas after 0 and 48h were measured by using the TScratch program (left panel). Graphs show relative values of unfilled areas and average values from a representative of multiple experiments performed in triplicate (right panel). Scale bar: 500 μm.

Table S1. PIEDA of YAP/TEAD1-IF2 complex (PDB ID: 3KYS).

YAP	TEAD1	PIE	ΔE^{es}	ΔE^{ex}	ΔE^{ct+mix}	ΔE^{di}	ΔG^{sol}
GLN053	GLU323	-8.729	-1.046	0.974	-1.279	-2.666	-4.711
	PRO342	-3.622	-3.318	1.147	-0.890	-1.740	1.179
	MET343	-6.501	-5.282	0.320	-0.733	-1.057	0.250
ILE054	GLU323	-18.893	-15.645	2.155	-1.787	-3.002	-0.614
VAL055	LYS321	-10.195	-7.009	4.551	-2.807	-4.100	-0.830
	VAL322	-15.344	-17.188	6.964	-1.551	-3.435	-0.134
HIS056	GLU320	-18.294	-16.495	1.771	-2.341	-3.849	2.620
	LYS321	-10.482	-12.686	8.840	-2.650	-3.701	-0.287
VAL057	GLN317	-4.189	-4.725	0.002	-0.127	-0.225	0.887
	GLU320	-6.909	-8.873	4.047	-0.084	-2.752	0.752
	LYS321	-5.261	-3.958	0.000	-0.111	-0.378	-0.814
ARG058	PHE314	-7.616	-7.177	-0.001	-0.092	-0.094	-0.252
	VAL318	-19.979	-20.965	3.719	-2.540	-5.252	5.059
	VAL319	-9.985	-9.641	2.720	-0.529	-3.168	0.634
ASP064	SER313	-26.145	-30.322	13.059	-5.460	-4.371	0.949
	VAL318	-3.091	-0.483	4.614	-2.258	-3.567	-1.398
	TYR346	-4.424	-4.420	0.383	-0.391	-1.404	1.408
LEU065	TYR346	-7.591	-2.867	2.699	-1.819	-5.478	-0.126
GLU066	LYS353	-29.102	-66.129	0.819	-0.868	-1.839	38.915
LEU068	PHE314	-4.187	-1.649	3.060	-1.661	-4.061	0.124
PHE069	LYS353	-4.415	-1.510	6.139	-1.846	-5.334	-1.864
	VAL366	-3.817	-0.198	1.016	-1.373	-2.893	-0.370
	ASN369	-4.437	-4.162	-0.001	-0.015	-0.091	-0.168

All energies are in kcal/mol. The calculation was conducted at FMO-MP2/6-31G**/PCM level.

Table S2. PIEDA of YAP/TEAD1-IF3 complex (PDB ID: 3KYS).

YAP	TEAD1	PIE	ΔE^{es}	ΔE^{ex}	ΔE^{ct+mix}	ΔE^{di}	ΔG^{sol}
PRO081	PHE314	-3.688	-1.507	0.903	-0.777	-2.560	0.253
THR083	GLU368	-7.008	-3.879	-0.001	0.016	0.032	-3.175
VAL084	ASP249	-4.499	-8.282	-0.001	-0.085	-0.156	4.025
MET086	GLU368	-31.779	-28.614	2.853	-4.385	-4.955	3.321
ARG087	GLU368	-36.612	-55.978	0.007	-0.502	-0.375	20.236
	GLU393	-49.261	-77.663	0.290	-2.093	-1.593	31.798
ARG089	GLN246	-8.278	-9.157	0.218	-0.597	-1.364	2.622
	ILE247	-28.389	-26.897	3.414	-1.831	-3.238	0.163
	ASP249	-98.352	-132.793	44.817	-11.091	-7.788	8.503
	GLU368	-47.110	-51.853	-0.001	0.029	0.031	4.684
LYS090	ASP243	-20.327	-40.835	0.000	0.000	0.000	20.508
	GLN246	-10.459	-4.163	4.529	-1.904	-4.580	-4.341
LEU091	GLN246	-6.043	-4.770	0.415	-0.949	-1.666	0.928
PRO092	GLU240	-6.753	-4.098	0.687	-1.501	-1.745	-0.096
SER094	GLU240	-29.904	-40.142	16.443	-7.010	-4.940	5.744
	TYR406	-5.339	-12.983	16.686	-4.877	-4.309	0.145
PHE095	LYS274	-7.356	-3.740	3.175	-1.682	-5.287	0.178
PHE096	LYS274	-41.982	-40.107	6.243	-2.974	-4.115	-1.030
PRO098	TRP276	-4.585	-1.730	7.484	-2.340	-7.833	-0.166
	HIS404	-5.389	-5.439	1.500	-1.375	-2.033	1.958
PRO099	TRP276	-3.222	-2.336	0.686	0.691	-1.935	-0.329
	HIS404	-3.699	-8.042	13.645	-3.149	-6.834	0.681
GLU100	GLN402	-6.270	0.424	0.240	-0.559	-1.044	-5.331

All energies are in kcal/mol. The calculation was conducted at FMO-MP2/6-31G**/PCM level.

Table S3. PIEDA of BY03 and TEAD1 complex.

BY03	Total	ES	EX	CT	DI	SL
**SER313**	-3.215	-1.384	3.698	-1.524	-3.817	-0.188
**LYS316**	-3.056	3.233	0	0	0	-6.289
**TYR346**	-8.346	-2.109	5.063	-2.185	-8.768	-0.347
**MET347**	-5.701	-4.499	1.441	-1.011	-1.899	0.267
**LYS353**	-49.137	-46.843	7.541	-4.048	-9.278	3.491
**LEU354**	-4.123	-1.115	0.548	-0.976	-2.271	-0.309
**LYS355**	-3.121	-0.477	0.001	0.041	-0.158	-2.527
**VAL366**	-4.797	-1.583	2.07	-0.977	-4.549	0.242
**PHE370**	-4.935	-3.149	3.802	-1.545	-4.448	0.404

Table S3: PIE for BY03 bindings to TEAD1. All energies are in kcal/mol. The calculation was conducted at FMO-MP2/6-31G**/PCM level.

Table S4. PIEDA of BY01 and TEAD1 complex.

BY01	Total	ES	EX	CT	DI	SL
**SER313**	-3.256	-2.66	3.545	-1.111	-3.08	0.051
**TYR346**	-6.453	-2.229	0.987	-1.195	-3.717	-0.3
**PHE350**	-6.545	-2.138	4.805	-2.292	-7.112	0.192
**LYS353**	-7.821	-6.73	8.469	-2.648	-8.252	1.34
**LEU354**	-3.315	-0.235	1.431	-0.912	-3.069	-0.529
**VAL366**	-6.299	0.117	4.812	-1.929	-8.267	-1.033
**PHE370**	-6.599	-4.732	4.919	-1.324	-5.452	-0.01

Table S4: PIE for BY01 bindings to TEAD1. All energies are in kcal/mol. The calculation was conducted at FMO-MP2/6-31G**/PCM level.

Table S5. PIEDA of BY02 and TEAD1 complex.

BY02	Total	ES	EX	CT	DI	SL
**SER313**	-9.402	-6.959	4.157	-1.834	-4.233	-0.534
**LYS316**	-6.182	-3.318	0	0	0	-2.865
**TYR346**	-19.112	-11.561	9.457	-4.203	-12.483	-0.322
**MET347**	-3.911	-2.262	0.219	-0.84	-1.272	0.244
**PHE350**	-6.503	-1.149	4.618	-2.423	-8.186	0.637
**LYS353**	-15.301	-3.753	5.233	-2.419	-7.881	-6.48
**LEU354**	-3.585	-0.278	1.714	-1.009	-3.507	-0.505
**VAL366**	-3.56	-1.115	2.163	-0.74	-3.741	-0.126
**LEU367**	-3.586	-2.012	1.978	-0.935	-2.193	-0.424

Table S5: PIE for BY02 bindings to TEAD1. All energies are in kcal/mol. The calculation was conducted at FMO-MP2/6-31G**/PCM level.

Reference

1. Gebäck, T.; Schulz, M.M.P.; Koumoutsakos, P.; Detmar, M. TScratch: a novel and simple software tool for automated analysis of monolayer wound healing assays. *https://doi.org/10.2144/000113083* **2018**, *46*, 265–274, doi:10.2144/000113083.
